# Relationship between training supervision and evolution of the density of GPs: a 3-year cohort study on French cities between 2018 and 2021

**DOI:** 10.1186/s12960-022-00740-1

**Published:** 2022-05-12

**Authors:** Anas Taha, Sébastien Dawidowicz, Véronique Orcel, Thibault Puszkarek, Marc Bayen, Sabine Bayen

**Affiliations:** 1 Department of General Practice, Université Paris-Est Créteil, 94000 Créteil, France; 2 Maison de Santé Universitaire de Sucy-en-Brie, 5 rue Ingres, 94370 Sucy-en-Brie, France; 3 Department of General Practice, Université de Bretagne Occidentale, 29200 Brest, France; 4 Department of General Practice, Université de Lille, 59000 Lille, France

**Keywords:** General practice, Teaching, Health services accessibility, France, Cohort studies

## Abstract

**Context:**

There is an uneven distribution of general practitioners (GPs) across territories of developed countries leading to inequalities in access to health care. Countries are implementing incentive or coercive policies depending on the characteristics of their health system. Several studies suggest that the location of practical training may influence the location of GPs’ practices. The objective of this study is to investigate the existence of a relationship between training supervision and evolution of the density of GPs in French municipalities between 2018 and 2021.

**Methods:**

The evolution of the density of GPs in almost all French municipalities between 2018 and 2021 was followed up. A bivariate statistical analysis was carried out to look for a relationship between the evolution of the density of GPs and the number of training supervisors. Other bivariate analyses were carried out with other factors likely to influence the density of GPs, such as the existence of financial aid in the territory or the age of GPs. A multivariate analysis with all the significant variables in bivariate analysis was then carried out using the stepwise descending method.

**Results:**

A total of 34 990 (99.9%) French municipalities were included in the follow-up. Among these, 9427 (26.9%) had a GP and 3866 (11%) had a GP involved in the training supervision. The density of GPs in French cities decreased on average by 2.17% between 2018 and 2021. Territories without training supervisors decreased by 4.63% while those with at least one increased by 1.36% (*p* < 0.01). This significant relationship was also found in multivariate analysis.

**Conclusion:**

The training supervision is associated with a better evolution of density of GPs in French municipalities. This association persisted when other factors were considered. The results of this 3-year follow-up may lead us to consider the training supervision as a factor to regulate the distribution of GPs.

**Supplementary Information:**

The online version contains supplementary material available at 10.1186/s12960-022-00740-1.

## Introduction

Many developed countries have inequalities in the distribution of general practitioners (GPs) across their territory [1]. Determinants of this distribution are multifactorial and depend either on territorial characteristics (infrastructure and equipment) or on physicians’ characteristics (availability, academic training, and personal history). It appears that physicians prefer urban settings to rural areas [2]. To regulate this distribution, countries implemented incentive or coercive policies depending on their health system characteristics [3]. Incentive measures include financial aid for installation, recruitment during the postgraduate medical training, recruitment of foreign doctors, promotion of a group medical practice (in health centres or multiprofessional practices) or residency training settings in areas with low density of physicians [4].

These trends are also observed in France: French public authorities implement territorial policies to attract and/or retain GPs in the most deprived areas [5, 6].

The recent decline in the number of trained GPs has led to a 9% decrease in the total number of physicians between 2010 and 2020. Forecasts predict this decline could continue until 2025 [7]. Evolution of the demography of GPs may increase the risk of worsening inequalities in the distribution of GPs across the country.

The uneven distribution of GPs in France is even more harmed by a health system poorly primary care-orientated [8], despite repeated calls from international organisations [9, 10]. In this context, the lack of GPs can lead patients to consult in secondary or tertiary level of care services (specialist doctors and hospitals), generating ineffective care pathways for both patients and health system [11].

Several studies found a relationship between location of the postgraduate medical training and subsequent practice in the same geographical area, suggesting the role of academic training as an instrument to regulate the distribution of physicians [12–14]. Postgraduate medical training in ambulatory and community settings seemed preferable to hospital-based training to promote primary care practice [15, 16]. But the level of evidence is intermediate. In France, medical students choose their medical discipline at the end of graduation. During postgraduation, general practice residents alternate training courses either in tertiary care in hospitals or in primary care. For the latter, residents are trained in general practice structures by training supervisors (TS). Most of TS are self-employed or salaried GPs. Their role is to supervise postgraduate students in general practice education. Between 2015 and 2020, the number of TS increased by 40.7% [17, 18]. More details about the organisation of training supervision in France are available in Additional file 1. Even if training supervision could be a potential tool to fight against territorial inequalities in GPs density, no cohort study has evaluated its effect on the evolution of the density of GPs across territories, especially in France.

The objective of this study is to investigate the existence of a relationship between training supervision and evolution of the density of GPs per capita in French municipalities.

## Methods

We conducted a prospective cohort study in French municipalities. The outcome was the evolution of the density of GPs per 10 000 inhabitants. Follow-up was carried out from 1 January 2018 to 1 January 2021. Year 2018 was chosen because it was the date with available data for TS. Municipalities were selected as territorial unit because it was the territorial scale having the most available data for confounders.

A database was set up. It gathered for each French municipality: evolution of the density of GPs per 10 000 inhabitants between 2018 and 2021, number of training supervisors, and other factors supposed to influence the evolution of the density of GPs.

This database was created by gathering data from 3 sources: the Regional Health Agency (RHA) and the French National Institute for Statistics and Economic Research (FNISER) registries and the National Union of Teachers of General Practice (NUTGP). All French municipalities were eligible for follow-up. Municipalities with missing data were not included.

With the RHA registry, we could extract data related to the evolution of the density per 10 000 inhabitants, the age range of GPs, the number of pharmacies, hospitals, health centres or multiprofessional practices (with either salaried or self-employed remuneration), and the existence or not of financial aid for medical activity. Financial aid for medical activity was defined by the RHA for each municipality. With the FNISER registry, we could extract data related to the urban or non-urban status of the territory according to their definition. The NTUGP provided the number of TS in each municipality. These data came from a national declarative survey in all the 35 French departments of general practice in 2018.

Only municipalities with at least one GP on 01/01/2018 were included in the statistical analysis. This database was registered to the French Data Protection Authority (Commission Nationale de l'Informatique et des Libertes, CNIL) in accordance with the current legislation.

From these data, the overall characteristics of GPs and TS were described as means and standard deviations or medians and interquartile ranges. The density of GPs per 10 000 inhabitants on 1 January 2018 and on 1 January 2021, and the evolution of the density between these two dates, were calculated. Because of the uneven distribution of French GPs across the country, no matching was possible in terms of size of the municipality. The outcome was defined as the evolution of the density of GPs per 10 000 inhabitants between 2018 and 2021. After assessing the normal distribution, a bivariate analysis was performed. A multivariate analysis using the backward stepwise selection was performed, retaining all variables with a *p* < 0.2 in the bivariate analysis. The correlations of the variables with each other were performed by Pearson’s test. Highly correlated variables were not included in the multivariate statistical model. Statistical analyses were conducted with STATA® version 12.

## Results

### Characteristics of the studied municipalities

Among the 35 011 French municipalities registered on 1 January 2018, data from 34 990 municipalities were collected. Data from 18 municipalities on the island of Mayotte and 3 others were missing.

As of 1 January 2018, 59,660 GPs were practising and were distributed in 9427 (26.9%) municipalities. The cumulative population of these municipalities was 55 813 568 inhabitants, representing 83.9% of the French population. A total of 9416 GPs were practising as TS. These GPs practised in 3866 (41.0%) municipalities. The median number of GPs in the municipalities with at least one GP was 3 [1; 5]. The median number of GPs in the endowed municipalities was 1 [1; 3]. In total, 1984 (21.1%) municipalities provided financial aid to GPs. The main characteristics of the municipalities are summarised in Table 1.

**Table 1:** Characteristics of French municipalities with at least one general practitioner on 01/01/2018

	*n*	Mean	St. deviation
General practitioners	59 660	6.33	18.51
Training supervisors	9416	0.99	2.69
Training supervisors’ density per 10 000 inhabitants	–	2.78	6.31
Multiprofessional practices	1043	0.11	0.33
Health centres	852	0.09	0.55
Pharmacies	20 239	2.23	5.72
Hospitals	1720	0.19	0.8

### Evolution of the density of general practitioners and training supervisors

In 2021, the overall medical density of GPs decreased by 2.17% compared to 2018 for all the included municipalities. There were 3866 municipalities with a practising TS and 5561 without.

The density of GPs per 10 000 increased by 1.36% in municipalities with practising residents and decreased by 4.63% in other municipalities. This difference was statistically significant at the t-test (*p* < *0.01*).

### Bivariate analysis

The evolution of medical density from 2018 to 2021 was analysed with other identified variables. A relationship was found between a favourable evolution of the density and the presence of TS in municipalities, the existence of multiprofessional practices on the territory and the proportion of GPs under 40 s in the municipality. A relationship was found between an unfavourable evolution of the density and the existence of financial aid or having GPs over 60 s.

Tables 2 and 3 summarise the results of the bivariate analyses according to the type of test (*t*-test or linear regression).

**Table 2:** Bivariate analysis with the evolution of municipal medical density between 2018 and 2021 (*t*-test)

	Yes		No		*p*
	*n*	Density per capita, 3 years evolution	*n*	Density per capita, 3 years evolution	
Financial support	1984	− 4.56%	7443	− 1.54%	< 0.01
Multiprofessional practices	1009	1.17%	8418	− 2.58%	< 0.01
Pharmacies	7809	0%	1618	− 12.91%	< 0.01
Health centres	478	− 4.63%	8949	− 2.00%	0.18
Hospital	1019	− 4.78%	8408	− 1.86%	0.03
Urban area	5589	0.48%	3838	− 6.04%	< 0.01

**Table 3:** Bivariate analysis with the evolution of municipal medical density between 2018 and 2021 (linear regressions)

Evolution of medical density from 2018 to 2021	Beta	95% CI		*p*
		Lower limit	Upper limit	
Density of general practitioners per 10 000 inhabitants	− 0.29	− 0.36	− 0.22	< 0.01
Proportion of general practitioners under 40 years	0.18	0.14	0.21	< 0.01
Proportion of general practitioners between 40 and 49 years	0.05	0.01	0.08	< 0.01
Proportion of general practitioners between 50 and 54 years	0.02	− 0.01	0.06	0.21
Proportion of general practitioners between 55 and 59 years	0.05	0.03	0.08	< 0.01
Proportion of general practitioners over 60 years	− 0.18	− 0.2	− 0.15	< 0.01
Proportion of women among general practitioners	0.03	0.01	0.06	0.02
Number of hospitals	− 0.86	− 1.9	0.19	0.11
Number of care homes	3.27	0.72	5.82	0.01
Number of pharmacies	0.02	− 0.13	0.16	0.8
Number of health centres	− 0.61	− 2.13	0.9	0.43

### Multivariate analysis

Multivariate linear regression was performed between the evolution of medical density from 2018 to 2021 and significant variables in bivariate analysis.

Using a backward stepwise selection, no longer significant variables were removed, until only significant variables remained. Table 4 shows the result of the multivariate analysis with the remaining significant variables.

**Table 4:** Evolution of medical density as a dependent variable (multivariate linear regression)

Evolution of medical density from 2018 to 2021	*Beta*	95% CI		*p*
		Lower limit	Upper limit	
Presence of training supervisors	4.12	2.38	5.85	< 0.01
Density in general practitioners per 10 000 inhabitants in 2018	− 0.39	− 0.47	− 0.31	< 0.01
Proportion of general practitioners between 40 and 49 years	− 0.12	− 0.16	− 0.07	< 0.01
Proportion of general practitioners between 50 and 54 years	− 0.13	− 0.18	− 0.09	< 0.01
Proportion of general practitioners between 55 and 59 years	− 0.1	− 0.14	− 0.06	< 0.01
Proportion of general practitioners over 60	− 0.26	− 0.29	− 0.22	< 0.01
Proportion of women among general practitioners	− 0.03	− 0.06	− 0.002	0.03
Urban municipality	2.98	1.14	4.81	< 0.01
Presence of a pharmacy	9.92	7.65	12.19	< 0.01
Presence of a hospital	− 5.83	− 8.54	− 3.12	< 0.01

## Discussion

Training supervision and presence of at least one pharmacy in the municipality were the only factors associated with a favourable evolution of the density of GPs in this 3-year follow-up study. Multivariate analysis found several other factors associated with a negative evolution of medical density. These factors had already been identified in the literature, such as rural territories [19], advanced age of doctors retiring earlier and existence of a significant basic medical density on the territory [20].

It should be noted that the proportion of women was associated with an unfavourable evolution in multivariate analysis, whereas it was related to a favourable evolution in bivariate analysis. The authors believe that this surprising result is due to a very unequal distribution of women in the population age pyramid.

Financial aid for installation was associated with an unfavourable evolution in bivariate analysis, but this relationship disappeared in multivariate analysis. This result is likely associated with a correlation between financial aid and rural territories. The latter is known to be associated with an unfavourable evolution of the density of GPs and it was confirmed in our work. It is interesting to note that a financial aid failed to counterbalance this effect. Since this grant is recent, we suggest that a follow-up is too short to bring out an effect on demography in our study.

The relationship between a favourable evolution of medical density and existence of multiprofessional practices disappeared in multivariate analysis. While group exercise is a favourable element in recruiting and keeping health professionals in a territory [21], we believe that this result is due to a relationship between presence of multiprofessional practices and density of TS in municipalities [22].

The relationship between presence of TS and favourable evolution of medical demography was highlighted by a significant coefficient in a cohort with only 3 years of follow-up. These elements tend to indicate the attractive feature of TS in their territory. However, this work only studied the effect of TS on their municipality of practice. It does not consider installations on the outskirts of their location of practice, which would have been a more relevant territorial unit to analyse the evolution of medical demography. Unfortunately, this choice was not possible for the methodological reasons mentioned above, but several studies suggest that new doctors also settle in neighbouring territories.

The issue of causality remains central. Do TS improve the attractiveness of their territory or is it the territory that is attractive? This work cannot formally answer this question. To do so, a randomised interventional study would be required, but it is not possible with the actual lack of TS. However, the multivariate analysis carried out in this work, although it could not integrate all the factors associated with the evolution of medical demography, found a persistent relationship between training supervision and favourable evolution of the density of GPs across the country. In addition, in a study of 2009, TS did not seem to be installed in different areas than other GPs in terms of access to care [23]. This study suggests the existence of a specific effect of TS on medical demography.

### Strengths and limitations

Several international studies have analysed the factors explaining the attractiveness of municipalities with the difficulty of comparing different health systems. Until now, no cohort studies evaluated training supervision. The collection of a pool of TS directly conducted by the general practice departments ensured the reliability of the data about the numbers and locations of TS. This cohort study covers almost the entire French territory, which improves its validity.

However, there are several limitations to consider. First, this work does not take into account the evolution of GPs in cities that were not endowed in 2018. A total of 255 municipalities are in this situation and the authors believe that their number is negligible on the results of this work. Moreover, this work does not consider the TS who stopped and those who started their activity between 2018 and 2021. Their number grew by 17.2%, or 1.624 more TS over this period [18, 24]. The effect of these TS has not been considered. We believe that GPs who started their TS activity after the beginning of the follow-up did not have enough time to have an impact on the attractiveness of their territory and that their effect can be neglected. Several elements which probably had an impact on the attractiveness of TS, such as their seniority and the length of time they received students, were unavailable for this database and could not be analysed. The financial aid analysed in this work correspond to the current national support. Local financial aids sometimes proposed by some areas could not be considered.

### Perspectives

The evolution of medical demography is a major concern for the coming years. In France, as elsewhere, it is relevant not to limit the issue of distribution of GPs to the number of trained GPs [25]. The influence of TS on the medical demography of their territory could constitute a simple and effective response to the problems of access to care. This impact could improve support for the deployment of TS in territories and, more generally, for considering an additional interest in primary care internships in the curriculum of general practice residents. The modest resources invested in recruitment, remuneration and education of training supervisors must be weighed against the considerable resources invested for the attractiveness of areas lacking of doctors, for which effects are still poorly known and limited in time [3].

Given the impact of the training supervision on medical demography, it would be consistent to value territories by the existence of TS.

## Conclusion

The training supervision is associated with an improvement of the density of GPs in the municipality of practice. Training supervision seems to improve the attractiveness of a territory. This justifies considering the training supervision as a facilitating factor to regulate the distribution of GPs across the national territory.

## Supplementary Information

**Additional file 1. ** Description of the training supervision in France.

## Data Availability

The datasets generated and/or analysed during the current study are not publicly available due to the general data protection regulation, but are available from the corresponding author on reasonable request.
