# Supplementary material for: Relationship between training supervision and evolution of the density of GPs: a 3-year cohort study on French cities between 2018 and 2021
Source: Hum Resour Health. 2022 May 12;20:39. doi: 10.1186/s12960-022-00740-1 (PMC9097434; doi:10.1186/s12960-022-00740-1)
Supplement: Supplementary file 1 — Additional file 1. Description of the training supervision in France. [file 12960_2022_740_MOESM1_ESM.docx]

In France, the medical education of general practice is composed of courses at the university and practice-based training.

Historically, all medical students were trained in hospitals. But the mismatch between teaching and hospitals-based clinical situations encountered (tertiary care) led to the creation of a specific teaching and training courses in general practice, independently from hospitals. Specialty training in general practice were introduced in 1997 and are carried out in private practices, health centers or multiprofessionnal practices. Medical students in speciality training programmes of general practice are referred as general practice residents (1). While this specialty training introduces residents to general practice, they also train residents for their future profession. The training is carried out by general practitioners who were specifically trained to supervise students. Depending on the seniority and autonomy of the residents, training supervisors can directly involve residents in consultations with them or let residents do their consultations alone and supervise them afterwards. In France, the general practice residency lasts 3 years and corresponds to the third cycle of medical studies, from the7^th^ to the 9^th^ year of studies. It is composed of two mandatory 6-month training periods in general practice. The rest of the residency, composed of four other 6-month training periods, takes place in hospital settings (with a mixture of specialties, such as obstetrics and gynaecology, paediatrics, geriatrics or internal medicine, adult emergency medicine).

Any general practitioner (GP) practicing in France can become a training supervisor (TS). Before this, GPs must follow a specific training course, which is validated and carried out by academic teachers. Further training courses are offered to supervisors all along their career. TS are paid by government grants. They are affiliated with a medical school but not with a hospital. The number of training supervisors has been gradually increasing since the beginning of their national census in 2011 (2), which suggests the attractiveness of the practice. Among French general practitioners, the practice of training supervision is associated with a lower risk of burnout (3).

1. Bloy G. Transmission of Professional Knowledge in General Medicine: The Case of Internships with Practitioners. Rev Francaise Aff Soc. 2005;(1):101–25.

2. National Union of Teachers of General Practice press release. [11,000 university lecturers by 2020!] [Internet]. 2021. Available from: https://www.snemg.fr/uploads/media/default/0001/01/e470655a19eb770151873dee12bbb8d8078f7769.pdf

3. Dutheil F, Parreira LM, Eismann J, Lesage FX, Balayssac D, Lambert C, et al. Burnout in French General Practitioners: A Nationwide Prospective Study. Int J Environ Res Public Health. 2021 Nov 16;18(22):12044.

Additional file 1 : description of the training supervision in France
